# Comprehensive eye care for children in rural Bangladesh: community- and school-based service models

**Published:** 2022-09-20

**Authors:** Johurul Islam Jewel, Mohammad Muhit

**Affiliations:** Senior Program Manager: CSF Global, Dhaka, Bangladesh.; President: CSF Global, Dhaka, Bangladesh.

**Timely eye care for children in underserved regions was made possible through community- and school-based eye care programmes in Bangladesh.**

CSF Global, in collaboration with government and non-governmental agencies in Bangladesh, and with financial support from the United States Agency for International Development (USAID), designed and implemented two eye care programmes for children in the most underserved regions of Bangladesh. The programmes were implemented in the four northern districts of Kurigram, Lalmonirhat, Nilphamari, and Gaibandha, and the three coastal districts of Patuakhali, Bhola, and Barisal in 2014–2017. The programme areas included the remote *char* land or river islands of Rangpur division and the sea islands in the Bay of Bengal, in Barisal division.

**Figure:**
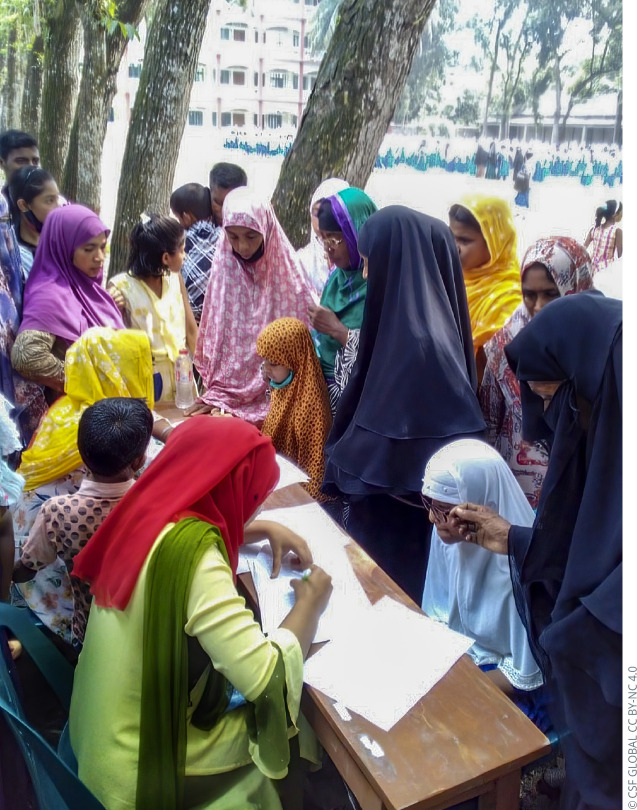
Examining Children in the community using key informant method. bangladesh

The programmes were based on two strategies:

- the key informant method (KIM) programme, which involves community-based screening
- a comprehensive school screening initiative.

## Key informant method programme

The programme team used the key informant method to identify children with blindness and severe visual impairment. These children are relatively few in number and often drop out of school. They also tend to live in remote rural communities, and have no access to sight-restoring cataract operations, low vision assistive devices such as white canes, visual rehabilitation (vocational training), or mobility training.

The team reached out to these children through community volunteers called **key informants** who underwent detailed training in how to identify children with visual impairments. They referred the children who were identified to nearby eye camps where an ophthalmologist carried out formal diagnostic assessments. Parents were recruited as key informants or referral ambassadors; they were also involved in disseminating information to raise eye health awareness.

The programme team established a referral network with eye hospitals, inclusive education centres, and rehabilitation centres, and referred children to these services, as needed. They also provided spectacles to children with low vision.

The programme identified 30,000 children who were blind or had severe visual impairment. A total of 500 children received vision-restoring cataract surgery, 1,000 received white canes and rehabilitation training, and 500 received low vision devices.

## Comprehensive school screening initiative

The comprehensive school screening initiative identified school children in remote areas with mild to moderate visual impairment due to uncorrected refractive error. There are, relatively, a larger number of children with these issues. Most of them are in regular schools in rural areas, where affordable and accessible eye and vision care services are limited or lacking.

School teachers and older students were trained to serve as vision screening volunteers. After screening, the volunteers made a list of children with vision impairment. These children were examined and given refraction. They received spectacle prescriptions, and were given appropriate spectacles by an optometrist who was available on call. Any child needing further examination by an ophthalmologist was referred to an eye hospital. Free spectacles were given to 12,000 children who were identified with refractive error during the programme period.

The long-term programme objective is to collect information to map children needing eye care services, and to develop and strengthen a network of key informants, government and non-government agencies, and ophthalmologists to provide treatment and rehabilitation services. The goal is to improve children's wellbeing, school attendance, academic performance, and overall functioning through timely eye care.

So far, 3,500 people have been trained to become key informants. Any committed member of the community can volunteer to be trained as a key informant.

Health communication leaflets, posters and billboards, face-to-face meetings, and seminars have been helpful in raising community awareness regarding comprehensive eye care for children. Increased awareness within the community is essential for the long-term sustainability of eye health programmes.

## Challenges

The following are some of the problems that were encountered:

- limited pool of people from which to select appropriate key informants
- restrictions on movement due to political unrest
- unavailability of specialised eye care services locally (i.e., in the programme area)
- access difficulties due to floods, especially during the monsoon (three to four months of the year).

**Figure:**
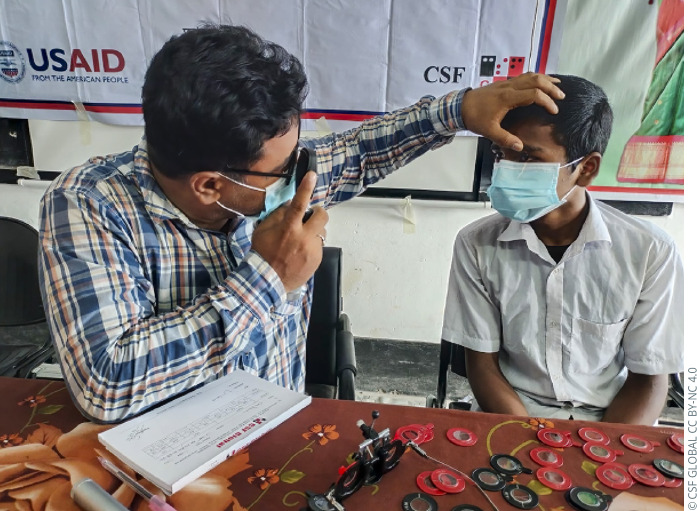
Examining children at school. bangladesh

To address the challenges, the team involved government health and education departments, non-governmental agencies, and local leaders in programme implementation. Importantly, the projects were run with community participation, which is the key to implementing a health programme covering a vast area and a large population in a short time and with limited resources.
